# Effects of sacubitril/valsartan on life quality in chronic heart failure: A systematic review and meta-analysis of randomized controlled trials

**DOI:** 10.3389/fcvm.2022.922721

**Published:** 2022-08-03

**Authors:** Yinyin Song, Zinan Zhao, Jingwen Zhang, Fei Zhao, Pengfei Jin

**Affiliations:** ^1^Department of Pharmacy, Beijing Hospital, National Center of Gerontology, Beijing, China; ^2^Institute of Geriatric Medicine, Chinese Academy of Medical Sciences, Beijing, China; ^3^Department of Pharmacy Administration and Clinical Pharmacy, Pharmaceutical Science, Peking University, Beijing, China

**Keywords:** sacubitril/valsartan, ACEI, ARB, health-related quality of life, heart failure, systematic review

## Abstract

**Aims:**

Sacubitril/valsartan has been demonstrated to have cardiovascular benefits in patients with chronic heart failure (CHF). We aimed to conduct a meta-analysis of its effects on life quality in patients with CHF, in comparison with the angiotensin-converting enzyme inhibitor/angiotensin receptor blocker (ACEI/ARB).

**Methods:**

PubMed, Embase, Cochrane Central Register of Controlled Trials (CENTRAL), and ClinicalTrials.gov were searched from inception through March 2022 for all relevant randomized controlled trials assessing the impact of sacubitril/valsartan and ACEI/ARB on health-related quality of life (HRQoL) in patients with CHF. Two reviewers independently conducted study selection, data extraction, and assessment of bias and quality of evidence. Review Manager 5.3 software was used for meta-analysis.

**Results:**

We included 10 clinical studies involving 10,426 patients with heart failure with reduced ejection fraction (HFrEF) and 7,689 patients with heart failure with preserved ejection fraction (HFpEF). Meta-analysis results showed that, in terms of the primary outcome, the sacubitril/valsartan group was superior than the ACEI/ARB group in improving HRQoL of HFrEF, and the difference was statistically significant (SMD 1.26; 95% CI: 0.14, 2.37; *p* = 0.03), while there was no significant difference between the two groups in HFpEF (SMD 0.37; 95% CI: −0.35, 1.09; *p* = 0.32). The effect of sacubitril/valsartan on the secondary outcome of the minimal important improvement rate of HRQoL in HFrEF was consistent with the primary outcome, while the effect in HFpEF was not clear. The descriptive analysis of individual studies indicated no significant difference in the improvement of 6-min walk distance between the two groups.

**Conclusion:**

Sacubitril/valsartan is beneficial to improve HRQoL outcome in patients with HFrEF with high quality of evidence. Compared with ACEI/ARB, sacubitril/valsartan was more effective. While in patients with HFpEF, this improvement was similar between the two groups.

## Introduction

Heart failure (HF) is a complex clinical syndrome in which symptoms result from a structural or functional cardiac disorder that impairs ventricular systolic and/or diastolic function. According to epidemiological data, the number of patients with HF worldwide was estimated to be 64 million in 2017 (1). With the aging of the population, the increased risk of cardiovascular disease, and the prolongation of the survival time of patients with HF, the prevalence of HF continues to rise, which is expected to increase by 25% in the next 20 years (2). The burden of symptoms and the disabling consequences of HF will affect the exercise capacity of patients and result in a decrease in the health-related quality of life (HRQoL), which significantly influences daily life. Compared with patients with other chronic diseases, the life quality of patients with HF is significantly reduced, which deserves more clinical attention (3, 4). In addition, improving life quality and maximizing function in daily life are increasingly recognized as one of the key targets of HF treatment. Therefore, we need to pay attention to the potential impact of drugs on life quality.

Sacubitril/valsartan as an angiotensin receptor-neprilysin inhibitor (ARNI) widely used in clinical treatment of HF can improve the imbalance between the renin-angiotensin-aldosterone system (RAAS) and natriuretic peptide system (5). The drug not only has significant advantages in the treatment of HF but also has potential benefits across a wide spectrum of cardiovascular disease, including hypertension, secondary prevention after acute myocardial infarction, arrhythmias, and so on (6–8). Some studies reported that sacubitril/valsartan could affect the risk of ventricular tachyarrhythmia in patients of heart failure with reduced ejection fraction (HFrEF) (5, 9). As the research progresses, sacubitril/valsartan has increasingly become a research hotspot in the therapy of cardiovascular disease and may play an important role in the whole process management of cardiovascular event chain in the future.

Previous studies demonstrated that sacubitril/valsartan had good clinical benefits to patients with HFrEF and was superior to angiotensin-converting enzyme inhibitor/angiotensin receptor blocker (ACEI/ARB) in reducing all-cause and cardiovascular mortality, as well as the rate of HF hospitalization (10). As a result of its antifibrotic and antihypertrophic effect, sacubitril/valsartan also has reversal effects on cardiac remodeling in patients with HFrEF (11). At the level of hemodynamics, sacubitril/valsartan can induce the continuous improvement of left ventricular ejection fraction, tricuspid annular plane systolic excursion, and systolic pulmonary atrial pressure, with a reduction in the incidence of cardiac valvular insufficiency (12). In addition, the drug can provide additional renal benefits for patients with HF. Compared with ACEI/ARB, sacubatril/valsartan can induce the preservation of the residual renal function (13). Several other studies supported that sacubitril/valsartan was associated with the reduction of the apnea burden in patients with HFrEF and the improvement of blood glucose control in patients with CHF and diabetes (14, 15). In terms of safety, sacubitril/valsartan has a lower risk of renal impairment and severe hyperkalemia, and does not increase the risk of severe angioedema compared with ACEI/ARB, but the risk of hypotension is slightly higher (16).

Health-related quality of life, as one of the key targets of HF treatment, has less attention. Moreover, different results in previous studies were found. The effect of sacubitril/valsartan on HRQoL is still unclear. This study aimed to conduct a comprehensive quantitative analysis of the available evidence from published RCT to assess the impact of sacubitril/valsartan *vs.* angiotensin-converting enzyme inhibitor/angiotensin receptor blocker (ACEI/ARB) on the life quality in patients with CHF.

## Materials and methods

### Search strategy

We conducted a systematic search in PubMed, EMBASE, Cochrane Central Register of Controlled Trials (CENTRAL), and ClinicalTrials.gov for relevant studies from inception through March 2022. The studies were restricted to those reported in English with terms related to HF and life quality, including “heart failure,” “HF,” “heart decompensation,” “heart insufficiency,” “heart incompetence,” “entresto,” “sacubitril/valsartan,” “valsartan/sacubitril,” “sacubitril plus valsartan,” “valsartan plus sacubitril,” “sacubitril and valsartan sodium hydrate drug combination,” “LCZ696,” “angiotensin receptor/neprilysin inhibit*,” “ARNI*,” “neprilysin inhibit*,” “randomized controlled trial,” “RCT,” “controlled clinical trial,” “random*,” “placebo,” “trial,” etc. The detailed search strategies for all databases are presented in Supplementary Table 1. We also tracked references of included studies or related systematic reviews to identify other potentially eligible studies.

### Study selection

Identified studies met the following inclusion criteria: (1) patients with CHF; (2) comparing sacubitril/valsartan with ACEI/ARB; (3) reporting data about HRQoL during follow-up; and (4) randomized, placebo-controlled clinical trials or its subgroup analysis. The exclusion criteria are as follows: (1) duplicate reports; (2) studies that did not provide enough data to analyze the primary outcome; and (3) studies of which the full text was not available.

### Study outcomes

The primary outcome was HRQoL data assessed by some HRQoL scales during the follow-up. The HRQoL scales are validated tools for assessing the life quality of patients. According to different testing purposes, it can be divided into universal scales and specific scales. The internationally recognized heart failure-specific HRQoL scales include the Kansas City Cardiomyopathy Questionnaire (KCCQ) and the Minnesota Living with Heart Failure Questionnaire (MLHFQ), and the universal scales include Short Form 36 (SF-36) and Nottingham Health Profile (NHP) (17). When several HRQoL scales were reported in the study, we preferred to use the disease-specific scales, which are more sensitive to the change of clinical status and more accurate in reflecting the direction and degree of change (18). For the KCCQ scale, the simplified version of KCCQ-12 has the same validity, reliability, responsiveness, and interpretability as KCCQ-23. When the study reported several KCCQ subscales, we preferred to extract the overall summary score, followed by the clinical summary score and total symptom score (17, 19). When the study reported outcomes at multiple timepoints, we selected the longest period of follow-up for analysis. The secondary outcomes were the minimal important improvement rate of HRQoL and the improvement of 6-min walk distance (6MWD) on the baseline. A 5-point increase or decrease of the KCCQ or MLHFQ score is generally considered to be a minimal but meaningful difference, which can reflect a clinically significant change in HF status. It has been demonstrated to be associated with other clinically important outcomes, such as the risk of death or hospitalization in HF (19).

### Data extraction, risk of bias, and quality of evidence assessment

Two reviewers independently conducted literature screening according to the inclusion and exclusion criteria, data extraction, and assessment of bias and quality of evidence. In case of disagreement, we consulted the corresponding author, and if necessary, we contacted the original author to determine the implementation process of the trial and to obtain missing data. We extracted the following data from each included study using a standardized data collection form: first author, publication year, demographic characteristics, number of patients, intervention control treatment, HRQoL outcome characteristics (scales, change from baseline), and follow-up duration. For studies that were reported repeatedly, we extracted the data from the most complete dataset for analysis. The risk of bias of the included studies was assessed using the Cochrane Collaboration Risk of Bias Tool, and the assessment contents included: (1) random sequence generation; (2) allocation concealment; (3) blinding of subjects and researchers; (4) blinding of outcome measurement; (5) incomplete outcome data; (6) selective outcome reporting, and (7) other bias (20). We graded quality of evidence for the primary outcome using the Grading of Recommendations Assessment, Development and Evaluation (GRADE) framework, which contains five downgrading factors: study limitation, inconsistency, indirectness, imprecision, and publication bias (21).

### Data synthesis and analysis

We used RevMan5.3 for data analysis. Since different scales were used to assess HRQoL, we presented continuous outcomes as SMD with 95% confidence intervals (CIs). For dichotomous outcomes, data were presented as risk ratio (RR) with 95% confidence intervals (CIs). We used *Z*-test to calculate whether the pooled effect was statistically significant (*p* < 0.05). The χ^2^ test was used to test heterogeneity. If there was no significant heterogeneity among the studies (*p* ≥ 0.10, *I*^2^ < 50%), we used the fixed-effect model. If the heterogeneity among the studies was large (*p* < 0.10, *I*^2^ ≥ 50%), we used the random-effect model, and analyzed the causes of heterogeneity (22). When there was significant heterogeneity, after excluding the errors of data extraction, we considered subgroup analysis if the relevant information of the original study was available, and then combined the results of sensitivity analysis to find the source of heterogeneity. If the reported studies for an outcome was less than 3 or there was unexplained heterogeneity, we conducted a descriptive analysis of the results of individual studies. Publication bias was assessed by using Begg’s test and Egger’s test. *P* < 0.05 was considered statistically significant.

## Results

### Study selection and characteristics

**FIGURE 1:**
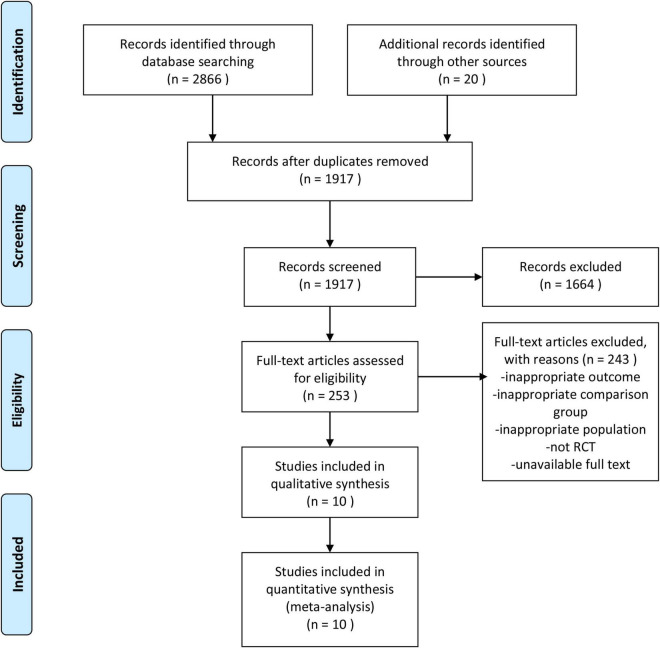
Flow diagram of study selection.

A total of 2,886 related studies were retrieved. After excluding duplicates, 1,917 studies remained. After screening the titles and abstracts, 1,664 studies were rejected for relevance. After reviewing the full text, 243 studies were excluded according to the inclusion and exclusion criteria, and 10 trials were finally included in our meta-analysis, involving 10,426 patients with HFrEF and 7,689 patients with heart failure with preserved ejection fraction (HFpEF) (10, 23–31). The study selection process is shown in Figure 1. The basic information of the included studies is shown in Table 1.

**TABLE 1:** Basic characteristics of included studies.

Study	Year	Type of HF	Sample size		Male/Female		Age, y		Intervention		NYHA class II/III/IV, %	Mean LVEF,%	Follow-up	HRQoL instrument
			
			ARNI	Control	ARNI	Control	ARNI	Control	ARNI	Control				
PARADIGM	2014	HFrEF	4209	4233	3330/879	3280/953	63.78 ± 11.52	63.82 ± 11.25	Sacubitril-valsartan (Target dose, 200 mg twice daily)	Enalapril (Target dose, 10 mg twice daily)	70.4/24.0/0.7	29.5	32 w	KCCQ CSS
EVALUATE-HF	2019	HFrEF	231	233	170/61	185/48	67.8 ± 9.8	66.7 ± 8.5	Sacubitril-valsartan (Target dose, 200 mg twice daily)	Enalapril (Target dose, 10 mg twice daily)	67.5/21.6/0.0	33.5	12 w	KCCQ OSS
AWAKE-HF	2021	HFrEF	70	70	52/18	56/14	62.3 ± 8.8	64.2 ± 11.6	Sacubitril-valsartan (Target dose, 200 mg twice daily)	Enalapril (Target dose, 10 mg twice daily)	90.0/10.0/0.0	30.9	8 w	KCCQ OSS
PARALLEL-HF	2021	HFrEF	112	113	96/16	96/17	69.0 ± 9.7	66.7 ± 10.9	Sacubitril-valsartan (Target dose, 200 mg twice daily)	Enalapril (Target dose, 10 mg twice daily)	92.0/4.5/0.0	28.1	24 w	KCCQ CSS
OUTSTEP-HF	2021	HFrEF	309	310	238/71	249/61	67.16 ± 11.04	66.62 ± 10.45	Sacubitril-valsartan (Target dose, 200 mg twice daily)	Enalapril (Target dose, 10 mg twice daily)	52.2/47.2/0.7	NA	12 w	SF-36 physical function subscale
ACTIVITY-HF	2021	HFrEF	103	98	86/17	77/21	66.1 ± 10.8	67.6 ± 10.0	Sacubitril-valsartan (Target dose, 200 mg twice daily)	Enalapril (Target dose, 10 mg twice daily)	0.5/99.5/0.0	31.9	12 w	KCCQ OSS
LIFE-HF	2022	HFrEF	167	168	120/47	125/43	60.2 ± 13.4	58.3 ± 13.1	Sacubitril-valsartan(Target dose, 200 mg twice daily)	Valsartan (Target dose, 160 mg twice daily)	22.4/40.9/34.0	20.4	24 w	KCCQ OSS
PARAGON	2019	HFpEF	2419	2403	1241/1178	1238/1165	72.7 ± 8.3	72.8 ± 8.5	Sacubitril-valsartan (Target dose, 200 mg twice daily)	Valsartan (Target dose, 160 mg twice daily)	77.3/19.4/0.4	57.6	32 w	KCCQ CSS
PARAMOUNT	2012	HFpEF	152	149	64/88	67/82	70.9 ± 9.38	71.2 ± 8.94	Sacubitril-valsartan (Target dose, 200 mg twice daily)	Valsartan (Target dose, 160 mg twice daily)	79.4/19.9/0.0	58	36 w	KCCQ OSS
PARALLAX	2021	HFpEF	1281	1285	638/643	627/658	72.9 ± 8.4	72.4 ± 8.6	Sacubitril-valsartan (Target dose, 200 mg twice daily)	Individualized medical therapy (1)Valsartan (Target dose, 160 mg twice daily); (2)Enalapril (Target dose, 10 mg twice daily)	67.6/31.8/0.3	56.3	24 w	KCCQ CSS

HF, heart failure; ARNI, angiotensin receptor-neprilysin inhibitor; LVEF, left ventricular ejection fraction; NYHA, New York Heart Association; HRQoL, health-related quality of life; HFrEF, heart failure with reduced ejection fraction; HFpEF, heart failure with preserved ejection fraction; KCCQ, Kansas City Cardiomyopathy Questionnaire; OSS, overall summary score; CSS, clinical summary score; NA, data not available; SF-36, Short Form 36.

### Risk of bias assessment

**FIGURE 2:**
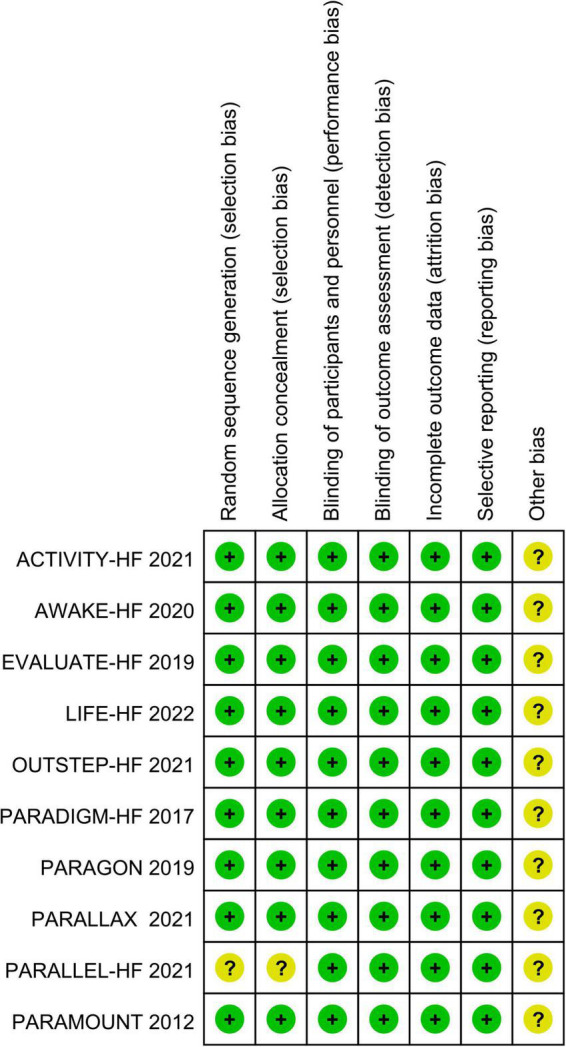
Risk of bias summary.

**FIGURE 3:**
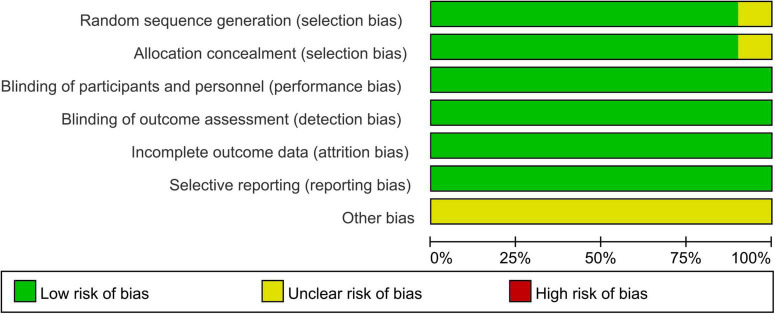
Risk of bias graph.

All included studies were randomized, double-blind, placebo-controlled trials. Nine studies reported detailed methods of random sequence generation and allocation concealment. One study only mentioned “random” and did not report how to implement allocation concealment. All included trials had a low risk of blinding, incomplete outcome data, and selective outcome reporting bias, while other biases were not clear. According to the Cochrane Handbook 5.1.0, the overall quality of the studies was high and the risk of bias was low. The results are shown in Figures 2, 3.

### Meta analysis results

#### The improvement of health-related quality of life from baseline

**FIGURE 4:**
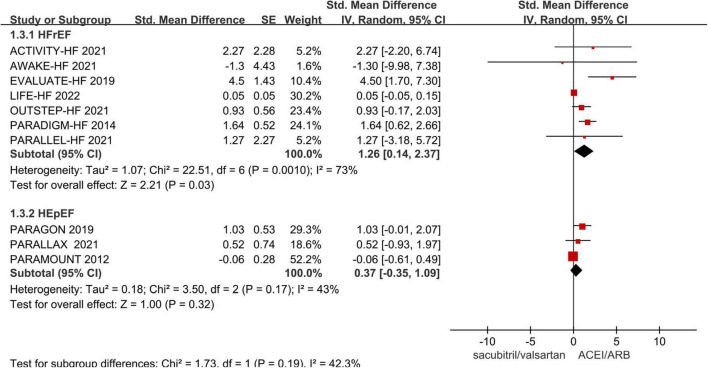
Meta-analysis forest plot of change in health-related quality of life (HRQoL) score.

Ten studies reported HRQoL data during follow-up. We performed subgroup analysis according to the phenotype of HF (HFrEF and HFpEF), and pooled effect value by random-effect model. The results demonstrated that sacubitril/valsartan group was superior than the control group in improving HRQoL of HFrEF, and the difference was statistically significant (SMD 1.26; 95% CI: 0.14, 2.37; *p* = 0.03). While in HFpEF, there was no significant difference between the two groups (SMD 0.37; 95% CI: −0.35, 1.09; *p* = 0.32), see Figure 4.

The heterogeneity among studies was high in the HErEF subgroup. We conducted sensitivity analysis to explore the source of heterogeneity, after excluding errors of data extraction. When the LIFE trial was excluded, the pooled effect value was still statistically significant. The effect of sacubitril/valsartan on HRQoL was similar with that of the overall analysis, but the heterogeneity among studies was significantly reduced (*I*^2^ = 17%, *p* = 0.30) (Supplementary Figure 1).

#### The minimal important improvement rate of health-related quality of life

Three trials compared the proportion of patients with an increase of ≥5 points on the KCCQ score of sacubitril/valsartan and ACEI/ARB in HErEF. The results of the heterogeneity test showed that there was statistical heterogeneity among studies (*p* < 0.10) and the heterogeneity could not be eliminated. Therefore, we performed a descriptive analysis of the results of individual trials without quantitative pooling. All trials showed that the minimal important improvement rate of HRQoL in the sacubitril/valsartan group was higher than that in the control group, including PARADIGM-HF trial (35% *vs.* 33%, *p* < 0.05), EVALUATE-HF trial (58% *vs.* 43%, *p* < 0.05), and PARALLEL-HF trial (17.3% *vs.* 13.2%, *p* < 0.05). Limited by the few studies, the evidence of the effect of sacubitril/valsartan *vs.* ACEI/ARB on the secondary outcome in HFrEF was not sufficient enough to draw a firm conclusion, but consistent with the direction of the primary outcome.

The other two trials reported this outcome between the two groups in HEpEF. In PARAGON-HF trial, more patients had a minimal important improvement in HRQoL at 8 months with sacubitril/valsartan than with ACEI/ARB (33.0% *vs.* 29.6%, *p* < 0.05), while in PARALLAX trial, there was no significant difference between the groups at 6 months (69.2% *vs.* 65.7%, *p* > 0.05).

#### The improvement of 6-minute walk distance from baseline

Two trials compared the change of 6MWD on the baseline of sacubitril/valsartan and ACEI/ARB in HErEF, and both showed negative results. In OUTSTEP-HF trial, the improvement of 6MWD from baseline between the two groups did not show statistical significance at 8 weeks (MD 8.87; 95% CI: −4.06, 21.80). In ACTIVITY-HF trial, there was also no significant difference at 12 weeks (MD 11.79; 95% CI: −7.02, 30.61).

One trial reported the change of 6MWD from baseline in HFpEF. The results showed that no significant difference in 6MWD among patients in HFpEF was observed between the groups at 24 weeks (MD −2.5; 95% CI: −8.5, 3.5).

### Sensitivity analysis and publication bias

The sensitivity analyses by excluding single study sequentially showed that when only trials with good homogeneity were pooled (except for LIFE trial), our result was robust (Supplementary Table 2).

Besides, we conducted a publication bias test for the outcome with more than five studies. For the primary outcome, Egger’s test showed a *p*-value of 0.054, and Begg’s test showed a *p*-value of 0.764, indicating no significant publication bias.

### Quality of evidence assessment

**FIGURE 5:**
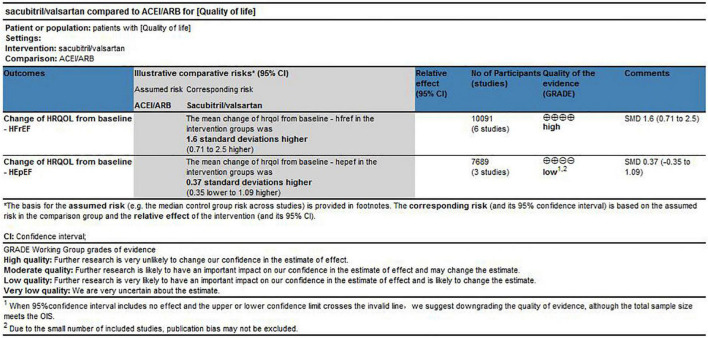
Quality of evidence for changes of HRQoL.

According to the GRADE framework, the quality of evidence that sacubitril/valsartan improved HRQoL scores superior to ACEI/ARB in HFrEF was improved after removing LIFE trial that contributed to heterogeneity between studies. While due to serious imprecision and the possibility of publication bias, the quality of evidence was downgraded to low for sacubitril/valsartan in HEpEF, see Figure 5.

## Discussions

This meta-analysis included 10 randomized, double-blind, placebo-controlled trials. The pooled effects demonstrated that the improvement of HRQoL score was greater of sacubitril/valsartan group than that of ACEI/ARB group in HFrEF, while no significant difference was found between the groups in HFpEF. This suggested that compared with ACEI/ARB, sacubitril/valsartan might have more advantages in improving HRQoL in HFrEF. Compared with the primary outcome, there were fewer clinical trials to assess the minimal important improvement rate of HRQoL, but the results from individual studies were consistent with the direction of primary outcome, which further confirmed that sacubitril/valsartan was more beneficial to HRQoL improvement in HFrEF. The exercise capacity of patients with HF was impaired, which had an important impact on daily activities. 6MWD is a commonly used index to assess exercise capacity of patients. The results showed that the impact of sacubitril/valsartan might be similar in improving 6MWD with ACEI/ARB. Heterogeneity analysis suggested that LIFE trial was the main source of heterogeneity. The control drug of the LIFE trial was valsartan, which was different from enalapril in other studies. In addition, the proportion of advanced heart failure in this population was relatively large, and NYHA (New York Heart Association) functional class IV accounted for about 34%, which was much higher than other studies. Therefore, we considered that the heterogeneity might be caused by the difference of the control group and NYHA functional class.

Chronic heart failure is a progressive disease that developed from abnormalities in the structure or function of the heart. The heart cannot supply enough blood to meet the metabolic needs of the body, which leads to the symptom burden such as dyspnea and fatigue. The progressive nature of HF and its symptom burden affects the quality of life in patients with HF, no matter in the physical or psychosocial aspects (32). Sacubitril/valsartan is the only ARNI on the market for the treatment of CHF, which can lead to dual inhibition of the RAAS and neprilysin and improve the therapeutic benefits of RAAS inhibitors alone. The drug can block the activation of RAAS, reduce the excitability of sympathetic nerve, relieve vasoconstriction, and reverse cardiac remodeling. Moreover, it can inhibit the activity of neprilysin to increase the levels of various endogenous vasoactive peptides, especially natriuretic peptides, so as to enhance the beneficial effect of RAAS blockade. Natriuretic peptides can promote diuresis, natriuresis, and vasodilation. It can also inhibit myocardial fibrosis and improve myocardial relaxation (33, 34). This meta-analysis suggested that sacubitril/valsartan had a good performance in improving HRQoL in HFrEF. Because the effects of the drug in blocking the activation of RAAS and increasing the sensitivity of natriuretic peptides may be translated into the clinically significant improvements in functional status. Therefore, the clinical symptoms of patients are improved and their exercise capacity is enhanced due to reduced cardiac load and improved cardiac function.

Patients with CHF often had functional limitations and impaired HRQoL. Improving the life quality is as important as prolonging the life. They are both the key targets of therapy in the management of patients with CHF (35). Therefore, it is of good clinical significance to assess the impact of the classic drugs, sacubitril/valsartan, and ACEI/ARB on HRQoL in patients with CHF. A previous study, PARADIGM-HF trial, showed that sacubitril/valsartan was superior to enalapril in improving HRQoL. However, the prospective comparative studies recently published about sacubitril/valsartan *vs.* ACEI/ARB, such as ACTIVITY-HF trial (1), PARALLEL-HF trial (1), and LIFE trial (1), showing that the differences of HRQoL between the two groups were negative. The results of existing clinical studies were contradictory. In addition, previous reviews had focused on the effect of sacubitril/valsartan on the outcomes of major cardiovascular events in HF, and less attention had been paid to HRQoL (36, 37). One systematic review assessed the impact of all contemporary drugs on HRQoL in HFrEF and included two randomized controlled trials about sacubitril/valsartan *vs.* enalapril, PARADIGM-HF trial and EVALUATE-HF trial (38). The study supported that sacubitril/valsartan was superior to enalapril, which was consistent with our study. Our study focused on a single drug, sacubitril/valsartan, to assess its impact on HRQoL in patients with CHF, and the number of included trials met the requirements for quantitative pooling, which could answer whether the life quality of patients with CHF could benefit from sacubitril/valsartan.

Our study was the first quantitative review of the impact of sacubitril/valsartan on HRQoL in patients with CHF from the perspective of evidence-based medicine. The results complemented the lack of evidence on the effect of sacubitril/valsartan in the treatment of CHF, clarified its beneficial effect on HRQoL in HFrEF, and provided more information for the clinical application of sacubitril/valsartan.

However, several potential limitations should be considered in our meta-analysis. First, there were some differences in follow-up duration among studies, which might influence our comparison to a certain extent. Nonetheless, the results of the pooled effect showed good consistency. Second, there were certain limitations in the selection of outcomes, which were mainly influenced by the reporting of the outcomes in studies. Third, we found significant heterogeneity among studies on some outcomes, which might result from the large differences in respect to control drugs, average dose, number of patients at different dose levels, and NYHA functional class among studies. Finally, although some studies reported that different population characteristics could have impacts on the treatment outcome with sacubitril/valsartan, such as the age and etiology differences, we did not conduct subgroup analysis (39, 40). Because there was lack of data concerning sacubitril/valsartan therapy with respect to different ages, etiologies of HF, NYHA functional classes, control drugs, duration of follow-up, etc.

## Conclusion

Sacubitril/valsartan was beneficial to improve HRQoL outcomes in patients with HFrEF with high-quality evidence. Compared with ACEI/ARB, sacubitril/valsartan was more effective. While in patients with HFpEF, this improvement was similar between the two classes of drugs. The effect of sacubitril/valsartan *vs.* ACEI/ARB on 6MWD was inconclusive. However, all included studies were not designed to show the impact of sacubitril/valsartan on HRQoL, but to use HRQoL as a secondary outcome or exploratory endpoint. Moreover, considering the limitations of our study, further trials are still needed to assess the effect of sacubitril/valsartan on HRQoL in patients with CHF to improve the evidence intensity.

## Author contributions

YS and ZZ were responsible for the literature search, data extraction, data analysis, and quality assessment and contributed to the study design. YS and JZ were responsible for the search strategy. YS was responsible for draft the manuscript. JZ, FZ, and PJ proofread and revised the manuscript. PJ was responsible for the project administration and funding acquisition. All authors contributed to the article and approved the submitted version.

## Abbreviations

ACEI: angiotensin-converting enzyme inhibitor
ARB: angiotensin receptor blocker
ARNI: angiotensin receptor-neprilysin inhibitor
HF: heart failure
CHF: chronic heart failure
HFrEF: heart failure with reduced ejection fraction
HFpEF: heart failure with preserved ejection fraction
HRQoL: health-related quality of life
KCCQ: Kansas City Cardiomyopathy Questionnaire
MLHFQ: Minnesota Living with Heart Failure Questionnaire
NHP: Nottingham Health Profile
SF-36: Short Form 36
6MWD: 6-minute walk distance
LVEF: left ventricular ejection fraction
NYHA: New York Heart Association
RAASi: renin-angiotensin-aldosterone system inhibitor
SMD: standard mean difference
MD: mean difference
RR: relative ratio
CI: confidence interval
GRADE: Grading of Recommendation AssessmentDevelopment and Evaluation
RCT: randomized controlled trial.

## References

[1] Luigi BN Zhong W Shu J Much AA Lotan D Grupper A Burden of heart failure and underlying causes in 195 countries and territories from 1990 to 2017. *Eur J Prevent Cardiol.* (2021). 28:1682–90. 10.1093/eurjpc/zwaa147 33571994

[2] Metra M Teerlink JR. Heart failure. *Lancet.* (2017) 390:1981–95. 10.1016/S0140-6736(17)31071-1 28460827

[3] Juenger J Schellberg D Kraemer S Haunstetter A Zugck C Herzog W Health related quality of life in patients with congestive heart failure: comparison with other chronic diseases and relation to functional variables. *Heart.* (2002) 87:235–41. 10.1136/heart.87.3.235 11847161 PMC1767036

[4] Hobbs F Kenkre JE Roalfe AK Davis RC Hare R Davies MK. Impact of heart failure and left ventricular systolic dysfunction on quality of life: a cross-sectional study comparing common chronic cardiac and medical disorders and a representative adult population. *Eur Heart J.* (2002) 23:1867–76. 10.1053/euhj.2002.3255 12445536

[5] Abumayyaleh M El-Battrawy I Behnes M Borggrefe M Akin I. Current evidence of sacubitril/valsartan in the treatment of heart failure with reduced ejection fraction. *Future Cardiol.* (2020) 16:227–36. 10.2217/fca-2020-0002 32186406

[6] Schmieder RE Wagner F Mayr M Delles C Ott C Keicher C The effect of sacubitril/valsartan compared to olmesartan on cardiovascular remodelling in subjects with essential hypertension: the results of a randomized, double-blind, active-controlled study. *Eur Heart J.* (2017) 38:3308–17. 10.1093/eurheartj/ehx525 29029087

[7] Pfeffer MA Claggett B Lewis EF Granger CB Køber L Maggioni AP Angiotensin receptor-neprilysin inhibition in acute myocardial infarction. *N Engl J Med.* (2021) 385:1845–55. 10.1056/NEJMoa2104508 34758252

[8] El-Battrawy I Borggrefe M Akin I. The risk for sudden cardiac death and effect of treatment with sacubitril/valsartan in heart failure. *JACC Heart Fail.* (2019) 7:999. 10.1016/j.jchf.2019.05.010 31672315

[9] El-Battrawy I Pilsinger C Liebe V Lang S Kuschyk J Zhou X Impact of sacubitril/valsartan on the long-term incidence of ventricular arrhythmias in chronic heart failure patients. *J Clin Med.* (2019) 8:1582. 10.3390/jcm8101582 31581623 PMC6832713

[10] McMurray J Packer M DeSai AS Gong J Lefkowitz MP Rizkala AR Angiotensin-neprilysin inhibition versus enalapril in heart failure. *N Engl J Med.* (2014) 371:993–1004. 10.1056/NEJMoa1409077 25176015

[11] Januzzi JL Prescott MF Butler J Felker GM Maisel AS McCague K Association of change in N-terminal Pro-B-type natriuretic peptide following initiation of sacubitril-valsartan treatment with cardiac structure and function in patients with heart failure with reduced ejection fraction. *JAMA.* (2019) 322:1085–95. 10.1001/jama.2019.12821 31475295 PMC6724151

[12] Abumayyaleh M Demmer J Krack C Pilsinger C El-Battrawy I Behnes M Hemodynamic effects of sacubitril/valsartan in patients with reduced left ventricular ejection fraction over 24 months: a retrospective study. *Am J Cardiovasc Drugs.* (2022). 10.1007/s40256-022-00525-w [Epub ahead of print]. 35353351 PMC9468101

[13] Spannella F Giulietti F Filipponi A Sarzani R. Effect of sacubitril/valsartan on renal function: a systematic review and meta-analysis of randomized controlled trials. *ESC Heart Fail.* (2020) 7:3487–96. 10.1002/ehf2.13002 32960491 PMC7754726

[14] Passino C Sciarrone P Vergaro G Borrelli C Spiesshoefer J Gentile F Sacubitril-valsartan treatment is associated with decrease in central apneas in patients with heart failure with reduced ejection fraction. *Int J Cardiol.* (2021) 330:112–9. 10.1016/j.ijcard.2021.02.012 33581182

[15] Wijkman MO Claggett B Vaduganathan M Cunningham JW Rørth R Jackson A Effects of sacubitril/valsartan on glycemia in patients with diabetes and heart failure: the PARAGON-HF and PARADIGM-HF trials. *Cardiovasc Diabetol.* (2022) 21:110. 10.1186/s12933-022-01545-1 35717169 PMC9206286

[16] Huang Y Zhang Y Ma L Zhou H Fang C Chen C. Adverse events of sacubitril/valsartan: a meta-analysis of randomized controlled trials. *J Cardiovasc Pharmacol.* (2021) 78:202–10. 10.1097/FJC.0000000000001049 33929386

[17] Kelkar AA Spertus J Pang P Pierson RF Cody RJ Pina IL Utility of patient-reported outcome instruments in heart failure. *JACC-Heart Fail.* (2016) 4:165–75. 10.1016/j.jchf.2015.10.015 26874386

[18] Spertus J Peterson E Conard MW Heidenreich PA Krumholz HM Jones P Monitoring clinical changes in patients with heart failure: a comparison of methods. *Am Heart J.* (2005) 150:707–15. 10.1016/j.ahj.2004.12.010 16209970

[19] Spertus JA Jones PG Sandhu AT Arnold SV. Interpreting the Kansas City cardiomyopathy questionnaire in clinical trials and clinical care. *J Am Coll Cardiol.* (2020) 76:2379–90. 10.1016/j.jacc.2020.09.542 33183512

[20] Higgins J Altman DG Gtzsche PC Jüni P Moher D Oxman AD The Cochrane collaboration’s tool for assessing risk of bias in randomized trials. *BMJ.* (2011) 11:343–51. 10.1136/bmj.d5928 22008217 PMC3196245

[21] Balshem H Helfand M Schünemann HJ Oxman AD Kunz R Brozek J GRADE guidelines: 3. Rating the quality of evidence. *Clin Epidemiol.* (2011) 64:401–6. 10.1016/j.jclinepi.2010.07.015 21208779

[22] Sedgwick P. Meta-analyses: what is heterogeneity? *BMJ.* (2015) 350:h1435. 10.1136/bmj.h1435 25778910

[23] Desai AS Solomon SD Shah AM Claggett BL Fang JC Izzo J Effect of sacubitril-valsartan vs enalapril on aortic stiffness in patients with heart failure and reduced ejection fraction: a randomized clinical trial. *JAMA J Am Med Assoc.* (2019) 322:1077–84. 10.1001/jama.2019.12843 31475296 PMC6749534

[24] Khandwalla RM Grant D Birkeland K Heywood JT Fombu E Owens RL The AWAKE-HF study: sacubitril/valsartan impact on daily physical activity and sleep in heart failure. *Am J Cardiovasc Drugs.* (2021) 21:241–54. 10.1007/s40256-020-00440-y 32978755

[25] Tsutsui H Momomura S Saito Y Ito H Yamamoto K Sakata Y Efficacy and safety of sacubitril/valsartan in Japanese patients with chronic heart failure and reduced ejection fraction - results from the PARALLEL-HF study. *Circ J Off J Japanese Circ Soc.* (2021) 85:584–94. 10.1253/circj.CJ-20-0854 33731544

[26] Piepoli MF Hussain RI Comin-Colet J Dosantos R Ferber P Jaarsma T OUTSTEP-HF: randomised controlled trial comparing short-term effects of sacubitril/valsartan versus enalapril on daily physical activity in patients with chronic heart failure with reduced ejection fraction. *Eur J Heart Fail.* (2021) 23:127–35. 10.1002/ejhf.2076 33314487

[27] Halle M Schöbel C Winzer EB Bernhardt P Mueller S Sieder C Randomized clinical trial on the short-term effects of 12-week sacubitril/valsartan vs. enalapril on peak oxygen consumption in patients with heart failure with reduced ejection fraction: results from the ACTIVITY-HF study. *Eur J Heart Fail.* (2021) 23:2073–82. 10.1002/ejhf.2355 34591356

[28] Mann DL Givertz MM Vader JM Starling RC Shah P McNulty SE Effect of treatment with sacubitril/valsartan in patients with advanced heart failure and reduced ejection fraction: a randomized clinical trial. *JAMA Cardiol.* (2022) 7:17–25. 10.1001/jamacardio.2021.4567 34730769 PMC8567189

[29] Solomon SD McMurray J Anand IS Ge J Lam C Maggioni AP Angiotensin–neprilysin inhibition in heart failure with preserved ejection fraction. *N Engl J Med.* (2019) 381:1609–20. 10.1056/NEJMc2000284 31475794

[30] Solomon SD Zile M Pieske B Voors A Shah A Kraigher-Krainer E The angiotensin receptor neprilysin inhibitor LCZ696 in heart failure with preserved ejection fraction: a phase 2 double-blind randomised controlled trial. *Lancet.* (2012) 380:1387–95. 10.1016/S0140-6736(12)61227-6 22932717

[31] Pieske B Wachter R Shah SJ Baldridge A Szeczoedy P Ibram G Effect of sacubitril/valsartan vs standard medical therapies on plasma NT-proBNP concentration and submaximal exercise capacity in patients with heart failure and preserved ejection fraction: the PARALLAX randomized clinical trial. *JAMA.* (2021) 326:1919–29. 10.1001/jama.2021.18463 34783839 PMC8596197

[32] Ponikowski P Voors AA Anker SD Bueno H Cleland JG Coats AJ 2016 ESC Guidelines for the diagnosis and treatment of acute and chronic heart failure: the Task Force for the diagnosis and treatment of acute and chronic heart failure of the European Society of Cardiology (ESC). Developed with the special contribution of the Heart Failure Association (HFA) of the ESC. *Eur J Heart Fail.* (2016) 18:891–975.1. 27207191 10.1002/ejhf.592

[33] Jessup M. Neprilysin inhibition — a novel therapy for heart failure. *N Engl J Med.* (2014) 371:1062–4. 10.1056/NEJMe1409898 25176014

[34] Hubers SA Brown NJ. Combined angiotensin receptor antagonism and neprilysin inhibition. *Circulation.* (2016) 133:1115–24. 26976916 10.1161/CIRCULATIONAHA.115.018622 PMC4800749

[35] McDonagh TA Metra M Adamo M Gardner RS Baumbach A Böhm M 2021 ESC Guidelines for the diagnosis and treatment of acute and chronic heart failure. *Eur Heart J.* (2021) 42:3599–726. 10.1093/eurheartj/ehab368 34447992

[36] Tromp J Ouwerkerk W Veldhuisen DJ Hillege HL Richards AM Meer P A systematic review and network meta-analysis of pharmacological treatment of heart failure with reduced ejection fraction. *JACC-Heart Fail.* (2021) 10:73–84. 10.1016/j.jchf.2021.09.004 34895860

[37] Charuel E Menini T Bedhomme S Pereira B Piñol-Domenech N Bouchant S Benefits and adverse effects of sacubitril/valsartan in patients with chronic heart failure: a systematic review and meta-analysis. *Pharmacol Res Perspect.* (2021) 9:e00844. 10.1002/prp2.844 34617669 PMC8495680

[38] Turgeon RD Barry AR Hawkins NM Ellis UM. Pharmacotherapy for heart failure with reduced ejection fraction and health-related quality of life: a systematic review and meta-analysis. *Eur J Heart Fail.* (2021) 23:578–89. 10.1002/ejhf.2141 33634543

[39] Abumayyaleh M El-Battrawy I Kummer M Pilsinger C Sattler K Kuschyk J Comparison of the prognosis and outcome of heart failure with reduced ejection fraction patients treated with sacubitril/valsartan according to age. *Fut Cardiol.* (2021) 17:1131–42. 10.2217/fca-2020-0213 33733830

[40] Abumayyaleh M Pilsinger C El-Battrawy I Kummer M Kuschyk J Borggrefe M Clinical outcomes in patients with ischemic versus non-ischemic cardiomyopathy after angiotensin-neprilysin inhibition therapy. *J Clin Med.* (2021) 10:4989. 10.3390/jcm10214989 34768510 PMC8584412

