# Supplementary material for: Effects of sacubitril/valsartan on life quality in chronic heart failure: A systematic review and meta-analysis of randomized controlled trials
Source: Front Cardiovasc Med. 2022 Aug 3;9:922721. doi: 10.3389/fcvm.2022.922721 (PMC9381951; doi:10.3389/fcvm.2022.922721)
Supplement: Supplementary file 2 [file Data_Sheet_1.docx]

Supplementary Material

**Supplementary Table 1.** The search strategies for all databases.

| **The search strategy for PubMed** | |
| --- | --- |
| **Number** | **Search terms** |
| #1 | "Sacubitril/valsartan"[MeSH Terms] |
| #2 | (((((((((((((((((((((((sacubitril plus valsartan[Title/Abstract]) OR (valsartan plus sacubitril[Title/Abstract])) OR (sacubitril/valsartan[Title/Abstract])) OR (valsartan/sacubitril[Title/Abstract])) OR (sacubitril-valsartan[Title/Abstract])) OR (sacubitril valsartan[Title/Abstract])) OR (sacubitrilvalsartan[Title/Abstract])) OR (sacubitril[Title/Abstract])) OR (Entresto[Title/Abstract])) OR (LCZ 696[Title/Abstract])) OR (LCZ696[Title/Abstract])) OR (LCZ-696[Title/Abstract])) OR (sacubitril valsartan sodium hydrate[Title/Abstract])) OR (sacubitril-valsartan sodium hydrate drug combination[Title/Abstract])) OR (sacubitril valsartan drug combination[Title/Abstract])) OR (sacubitril valsartan sodium anhydrous[Title/Abstract])) OR (sacubitril-valsartan sodium anhydrous drug combination[Title/Abstract])) OR (angiotensin receptor/neprilysin inhibit*[Title/Abstract])) OR (angiotensin receptor-neprilysin inhibit*[Title/Abstract])) OR (angiotensin receptor neprilysin inhibit*[Title/Abstract])) OR (angiotensin-neprilysin inhibition[Title/Abstract])) OR (ARNI[Title/Abstract])) OR (ARNIs[Title/Abstract])) OR (Neprilysin inhibit*[Title/Abstract]) |
| #3 | #1 OR #2 |
| #4 | "Heart Failure"[MeSH Terms] |
| #5 | (((((((((((((heart failure[Title/Abstract]) OR (heart decompensation[Title/Abstract])) OR (heart insufficiency[Title/Abstract])) OR (heart incompetence[Title/Abstract])) OR (cardiac failure[Title/Abstract])) OR (cardiac decompensation[Title/Abstract])) OR (cardiac insufficiency[Title/Abstract])) OR (cardiac incompetence[Title/Abstract])) OR (cardial decompensation[Title/Abstract])) OR (cardial insufficiency[Title/Abstract])) OR (decompensation, heart[Title/Abstract])) OR (myocardial failure[Title/Abstract])) OR (HF[Title/Abstract])) OR (chronic heart failure[Title/Abstract]) |
| #6 | #4 OR #5 |
| #7 | ((((((((randomized controlled trial[Publication Type]) OR (controlled clinical trial[Title/Abstract])) OR (randomized[Title/Abstract])) OR (placebo[Title/Abstract])) OR (drug therapy[MeSH Subheading])) OR (randomly[Title/Abstract])) OR (trial[Title/Abstract])) OR (groups[Title/Abstract])) NOT ((animals[MeSH Terms]) NOT (humans[MeSH Terms])) |
| #8 | #3 AND #6 AND #7 |
| **The search strategy for Embase** | |
| **Number** | **Search terms** |
| #1 | 'sacubitril plus valsartan'/exp |
| #2 | 'sacubitril plus valsartan':ab,ti OR 'valsartan plus sacubitril':ab,ti OR 'sacubitril-valsartan':ab,ti OR 'sacubitril valsartan':ab,ti OR 'sacubitrilvalsartan':ab,ti OR 'sacubitril':ab,ti OR 'entresto':ab,ti OR 'neparvis':ab,ti OR 'lcz 696':ab,ti OR 'lcz696':ab,ti OR 'lcz-696':ab,ti OR 'sacubitril valsartan sodium hydrate':ab,ti OR 'sacubitril-valsartan sodium hydrate drug combination':ab,ti OR 'sacubitril valsartan drug combination':ab,ti OR 'sacubitril valsartan sodium anhydrous':ab,ti OR 'sacubitril-valsartan sodium anhydrous drug combination':ab,ti |
| #3 | 'angiotensin receptor neprilysin inhibitor'/exp |
| #4 | 'angiotensin receptor-neprilysin inhibit*':ab,ti OR 'angiotensin receptor neprilysin inhibit*':ab,ti OR 'angiotensin-neprilysin inhibition':ab,ti OR 'arni':ab,ti OR 'arnis':ab,ti OR 'neprilysin inhibit*':ab,ti |
| #5 | #1 OR #2 OR #3 OR #4 |
| #6 | 'heart failure'/exp |
| #7 | 'heart failure':ab,ti OR 'heart decompensation':ab,ti OR 'heart insufficiency':ab,ti OR 'heart incompetence':ab,ti OR 'cardiac failure':ab,ti OR 'cardiac decompensation':ab,ti OR 'cardiac insufficiency':ab,ti OR 'cardiac incompetence':ab,ti OR 'cardial decompensation':ab,ti OR 'cardial insufficiency':ab,ti OR 'decompensation, heart':ab,ti OR 'myocardial failure':ab,ti OR 'HF':ab,ti OR 'chronic heart failure':ab,ti |
| #8 | #6 OR #7 |
| #9 | 'randomized controlled trial':ab,ti OR 'RCT':ab,ti OR 'controlled clinical trial':ab,ti OR 'random*':ab,ti OR 'placebo':ab,ti OR 'trial':ab,ti |
| #10 | #5 AND #8 AND #9 |
| **The search strategy for Cochrane Library** | |
| **Number** | **Search terms** |
| #1 | (sacubitril plus valsartan):ab,ti,kw OR (valsartan plus sacubitril):ab,ti,kw OR (sacubitril-valsartan):ab,ti,kw OR (sacubitril valsartan):ab,ti,kw OR (sacubitrilvalsartan):ab,ti,kw OR (sacubitril):ab,ti,kw OR (entresto):ab,ti,kw OR (neparvis):ab,ti,kw OR (LCZ 696):ab,ti,kw OR (LCZ696):ab,ti,kw OR (LCZ-696):ab,ti,kw OR (sacubitril valsartan sodium hydrate):ab,ti,kw OR (sacubitril-valsartan sodium hydrate drug combination ):ab,ti,kw OR (sacubitril valsartan drug combination):ab,ti,kw OR (sacubitril valsartan sodium anhydrous):ab,ti,kw OR (sacubitril-valsartan sodium anhydrous drug combination ):ab,ti,kw OR (angiotensin receptor-neprilysin inhibit*):ab,ti,kw OR (angiotensin receptor neprilysin inhibit*):ab,ti,kw OR (angiotensin-neprilysin inhibition):ab,ti,kw OR (ARNI):ab,ti,kw OR (ARNIs):ab,ti,kw OR (Neprilysin inhibit*):ab,ti,kw |
| #2 | MeSH descriptor: [Heart Failure] explode all trees |
| #3 | (heart failure):ab,ti,kw OR (heart decompensation):ab,ti,kw OR (heart insufficiency):ab,ti,kw OR (heart incompetence):ab,ti,kw OR (cardiac failure):ab,ti,kw OR (cardiac decompensation):ab,ti,kw OR (cardiac insufficiency):ab,ti,kw OR (cardiac incompetence):ab,ti,kw OR (cardial decompensation):ab,ti,kw OR (cardial insufficiency):ab,ti,kw OR (decompensation, heart):ab,ti,kw OR (myocardial failure):ab,ti,kw OR (HF):ab,ti,kw OR (chronic heart failure):ab,ti,kw |
| #4 | #2 OR #3 |
| #5 | (randomized controlled trial):ab,ti,kw OR (RCT):ab,ti,kw OR (controlled clinical trial):ab,ti,kw OR (random*):ab,ti,kw OR (placebo):ab,ti,kw OR (trial):ab,ti,kw |
| #6 | #1 AND #4 AND #5 |

**Supplementary Table 2.** Sensitivity analysis by excluding single studies sequentially.

| Study omitted | SMD (95%CI) |
| --- | --- |
| HFrEF |  |
| ACTIVITY-HF 2021 | 1.64(0.57, 2.70) |
| AWAKE-HF 2021 | 1.68(0.69, 2.68) |
| EVALUATE-HF 2019 | 1.32(0.59, 2.04) |
| OUTSTEP-HF 2021 | 1.99(0.94, 3.05) |
| PARADIGM-HF 2014 | 1.88(0.16, 3.59) |
| PARALLEL-HF 2021 | 1.70(0.62, 2.77) |
| HFpEF |  |
| PARAGON 2019 | 0.01(-0.50, 0.53) |
| PARALLAX 2021 | 0.39(-0.66, 1.44) |
| PARAMOUNT 2012 | 0.86(0.01,1.70) |

HFrEF, heart failure with reduced ejection fraction; HFpEF, heart failure with preserved ejection fraction; SMD, standardized mean difference; CI, confidence interval


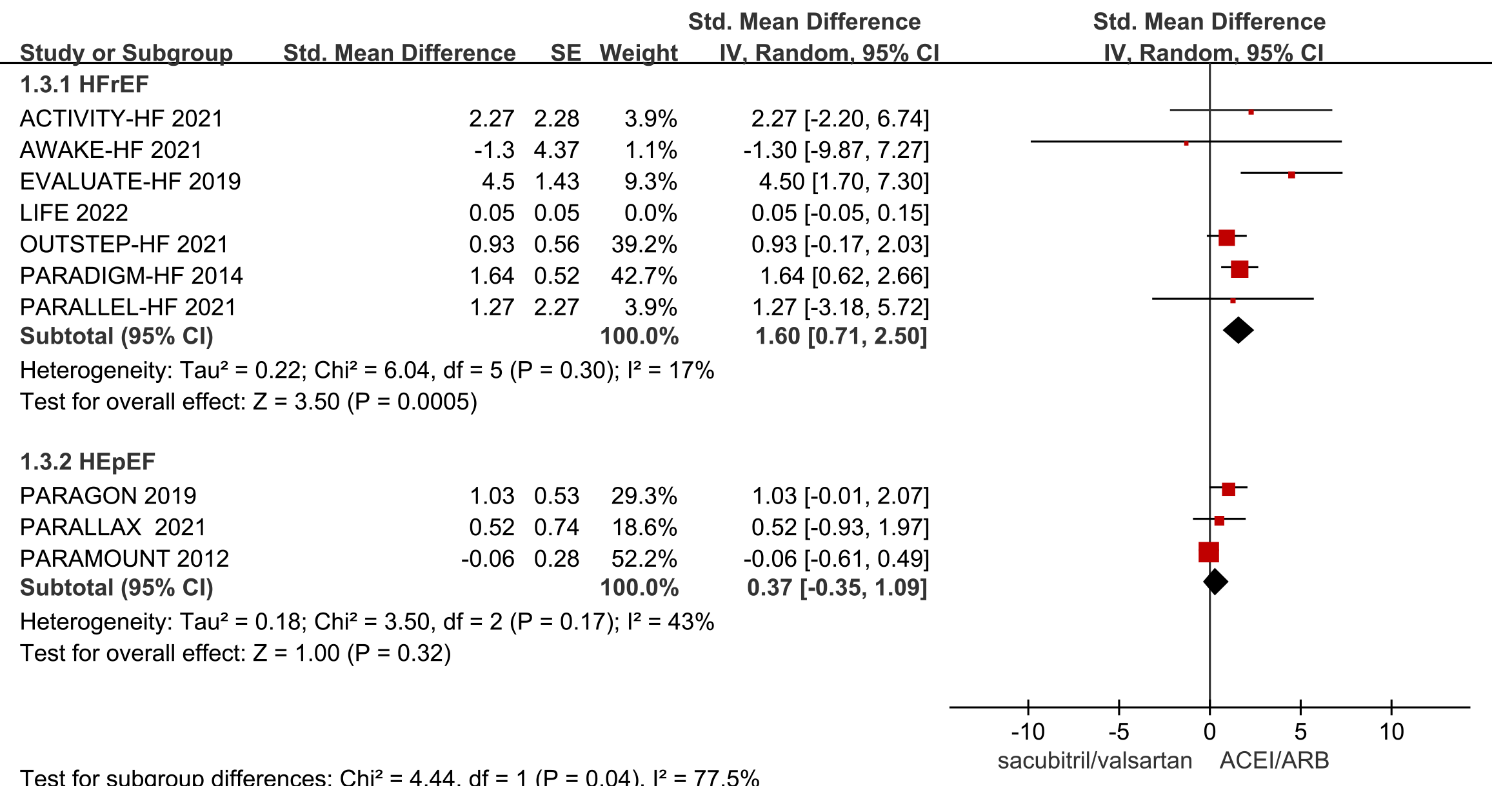


**Supplementary Figure 1.** Meta-analysis forest plot of change in HRQoL score without LIFE trial
